# Three-dimensional cardiac models: a pre-clinical testing platform

**DOI:** 10.1042/BST20230444

**Published:** 2024-05-23

**Authors:** Eline Groen, Christine L. Mummery, Loukia Yiangou, Richard P. Davis

**Affiliations:** 1 Department of Anatomy and Embryology, Leiden University Medical Center, Leiden, The Netherlands; 2 The Novo Nordisk Foundation Center for Stem Cell Medicine (reNEW), Leiden University Medical Center, 2300RC Leiden, The Netherlands

**Keywords:** cardiac disease, cardiac microtissue, cardiac organoids, engineered heart tissue, pluripotent stem cells, pre-clinical testing

## Abstract

Major advancements in human pluripotent stem cell (hPSC) technology over recent years have yielded valuable tools for cardiovascular research. Multi-cell type 3-dimensional (3D) cardiac models in particular, are providing complementary approaches to animal studies that are better representatives than simple 2-dimensional (2D) cultures of differentiated hPSCs. These human 3D cardiac models can be broadly divided into two categories; namely those generated through aggregating pre-differentiated cells and those that form self-organizing structures during their *in vitro* differentiation from hPSCs. These models can either replicate aspects of cardiac development or enable the examination of interactions among constituent cell types, with some of these models showing increased maturity compared with 2D systems. Both groups have already emerged as physiologically relevant pre-clinical platforms for studying heart disease mechanisms, exhibiting key functional attributes of the human heart. In this review, we describe the different cardiac organoid models derived from hPSCs, their generation methods, applications in cardiovascular disease research and use in drug screening. We also address their current limitations and challenges as pre-clinical testing platforms and propose potential improvements to enhance their efficacy in cardiac drug discovery.

## Introduction

Cardiovascular disease continues to be a leading cause of death globally, yet the mechanisms underlying cardiac diseases remain poorly understood and effective drug treatments are limited [1]. This knowledge and therapy gap is partly attributable to a paucity of physiologically-relevant human models. Primary human cardiac tissue is not easily accessible and is difficult to maintain in the laboratory, and animal models such as rodents, do not fully mimic human cardiac pathologies due to anatomical and size differences [2,3]. Hence, there is a pressing need for viable human cardiac models to improve our understanding of heart development and disease mechanisms, and for identifying potential targets for drug interventions.

Unlike the intestine [4], the heart lacks an endogenous stem cell population [5,6], precluding the generation of adult stem cell organoids from biopsies. However, human pluripotent stem cells (hPSCs), either as human embryonic stem cells (hESCs) or human induced pluripotent stem cells (hiPSCs), have become pivotal in cardiac research over the past two decades [7,8]. Coupled with now facile gene editing technologies and robust protocols for generating nearly all cell types of the body *in vitro,* these cells are offering increasingly deeper insights into human tissue physiology and disease [9,10].

**Figure 1.**
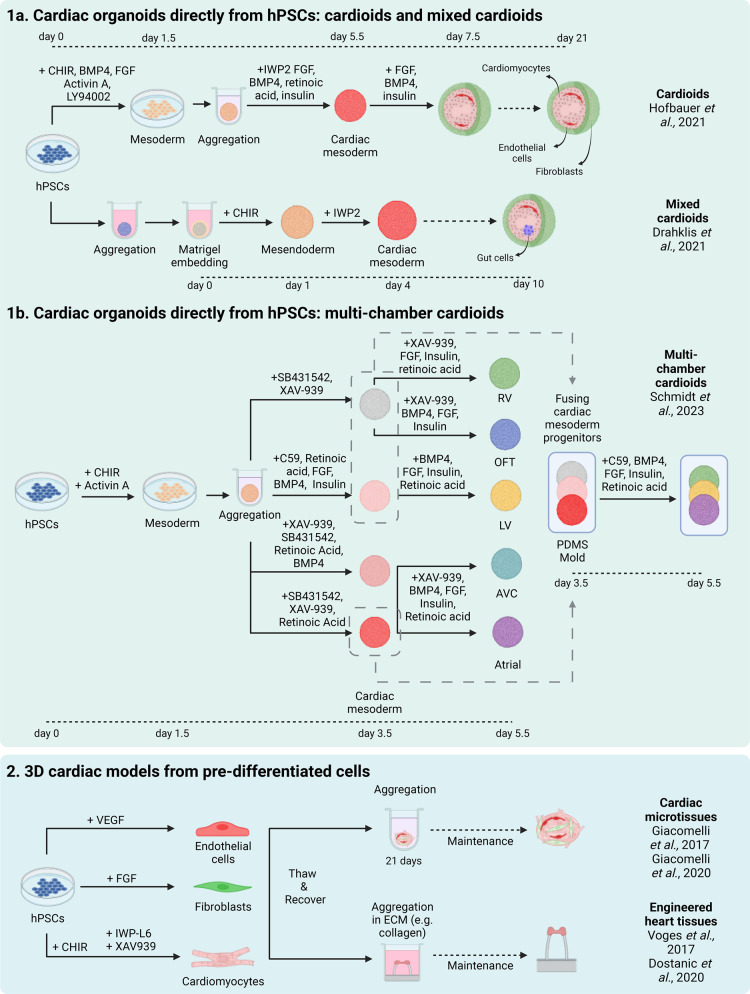
Schematic overview of protocols to develop 3D cardiac cellular models from hPSCs. (1) Generation directly from hPSCs. These cardiac organoid models include cardioids, mixed cardioids and multi-chamber cardioids. This process mainly involves manipulating the WNT signalling pathway. For cardioids and mixed cardioids, additional growth factors or Matrigel are used to further induce cardiac specification. To create compartment-specific cardioids, such as left ventricle (LV), right ventricle (RV), atrial, outflow tract (OFT), or atrioventricular canal (AVC), small adjustments are made to the concentrations of CHIR99021 and WNT inhibitors, along with supplementation of different concentrations of TGF-β inhibitor SB-431542, Retinoic acid, FGF and BMP4. These region-specific cardioids can be combined to form multi-chamber cardioids by fusing them at the cardiac mesoderm stage in a polydimethylsiloxane (PDMS) mold and further differentiating them in a co-development patterning medium. (2) Generation from pre-differentiated cardiac cell types. These models include EHTs and cMTs for which ECs, cFBs and CMs are differentiated from hiPSCs. The pre-differentiated cells can be cryopreserved, and then thawed and recovered for aggregation. For EHTs, the cells are combined with an ECM such as collagen to aid aggregation around pillars, while cMTs are aggregated in V-shaped or round-bottomed plates to support formation. The CMs in both EHTs and cMTs may undergo further maturation over time.

In cardiac research, 2-dimensional (2D) cultures of hPSC-derived contractile cardiomyocytes (hPSC-CMs) have been widely employed to investigate cardiac diseases, assess toxicity, and conduct drug screening [11–14]. However, these 2D models are mainly monotypic cultures and lack the cellular complexity and spatial interactions of real heart tissue. This is where in recent years, 3-dimensional (3D) 3D cardiac tissues derived from either undifferentiated hPSCs or differentiated hPSC-derivatives have significantly advanced the field by better replicating heart structure and function [15–19]. However, this speed of development has also sparked some controversies and debates regarding the definition and characteristics of what constitutes a cardiac organoid. Traditionally, organoids are defined as 3D structures grown from stem cells that replicate some of the key functional and structural characteristics of an organ [20]. Cardiac organoids, or cardioids as referred to by some in the field, are typically formed by aggregating hPSCs into spheroids, that are sometimes embedded in Matrigel, and inducing differentiation using the same developmental principles and similar cocktails of growth factors and small molecules to those employed in 2D cardiac cell differentiation [15,19,21,22] (Figure 1). This approach allows for effective and efficient self-organization within the cardiac organoids, with some strikingly recapitulating aspects of heart development, such as an organized composition of tissue layers.

Engineered heart tissues (EHTs) [17,18,23] and cardiac microtissues (cMTs) [16,24] represent another class of 3D cell models (Figure 1). These engineered models can be created by combining cell types such as cardiomyocytes (CMs), cardiac fibroblasts (cFBs) and endothelial cells (ECs), pre-differentiated from hPSCs using a combination of growth factors and small molecules to modulate WNT, TGF-β, and FGF signaling pathways for cell fate specification [25,26]. Alternatively, some of these models combine hPSC-CMs with primary cell-derived cFBs or ECs that have been isolated from tissue samples [27,28]. In the case of EHTs, the pre-differentiated cells are aggregated in an extracellular matrix (ECM) mix that may include collagen or Matrigel and cultured around flexible pillars. Within a few days they form elastic-band like structures around the pillars, allowing force measurements as a function of pillar displacement. Alternatively, ring-shaped EHTs can be generated by adding the ECM-cell mix in circular molds and placed on a silicon stretcher [29]. In contrast, cMTs are generated by aggregating the cells in multi-well plates where they form single spheroids [16–18,30,31].

Both EHT and cMT models often exhibit features like self-assembled endothelial networks and enhanced CM maturation, as evidenced by improved sarcomere organization, postnatal cardiac gene expression and more adult electrophysiological properties [24,32–34]. Cardiac organoids, on the other hand, can form more complex structures, including cavities, endothelial networks, as well as endocardium, myocardium and epicardium layers [15,18,19,21,23,35–37]. Some more complex organoid models even incorporate non-cardiac cell types, such as liver anlage, gut tube-like structures and innervation [38–40] (Figure 1).

In general, models derived from pre-differentiated cells are considered more suitable for modelling adult or late-stage disease due to their maturity and the relative cell composition can be well controlled. This makes the model highly reproducible and provides opportunities to mix both wildtype and disease cell types together. Cardioids, by contrast, are well-suited to mimic certain aspects of cardiac development, making them compatible for studying congenital heart defects (CHD) [21]. Along with modelling disease, these 3D cardiac models are being applied in drug discovery pipelines, with examples of EHTs and cMTs being used to screen compound libraries [23,28,41–43]. Cardiac organoids are a relatively new model and therefore there are few reports describing compound screens. However as the technology matures, they may become the preferred model when evaluating effects on cardiac development [44,45]. Additionally, improvements regarding standardization and automation will enhance the utility of all these models for high-throughput drug screening [46,47]. In the following sections, we will review the value and potential limitations of 3D cardiac models, focusing on the use of cardiac organoids/cardioids, EHTs and MTs in pre-clinical testing.

## Disease modelling

Numerous cardiac pathologies have been modeled using 3D cardiac cellular models, particularly EHTs and cMTs. These models have proven instrumental in studying a spectrum of conditions, including genetic cardiac diseases such as cardiomyopathies and cardiac arrhythmias, as well as myocardial infarction (MI).

MI, which globally is a leading cause of death [48], presents a pressing need for human *in vitro* models to identify potential treatments for post-MI pathology. These models will not only aid drug discovery, but also hold promise in developing regenerative therapies aimed at promoting CM proliferation. One strategy employed to simulate MI in 3D cardiac models, specifically cardioids and EHT-like constructs, has been cryoinjury [15,30], while other studies have used hypoxia in combination with noradrenaline to mimic the effects of MI in cMTs [49]. Both models exhibit MI-like responses, including significant cell death, fibrosis at the injury site, and elevated clinically relevant pathological markers like lactate dehydrogenase and troponin I in the culture medium [15,30,49] (Table 1). Notably, Voges et al. [30] observed complete functional recovery in their EHT-like constructs within 2 weeks, suggesting that the CMs retained a regenerative capacity that was most likely because they were still fetal-like and therefore were in the cell cycle or could be more readily stimulated to divide [30]. These studies underscore the potential of 3D cardiac models in mimicking clinical aspects of MI, although further maturation of CMs is likely required for observing longer-term MI effects.

**Table 1.** Studies using 3D cardiac systems to model cardiac disease

Disease	Model	Method	Metabolism	Fibrosis	Calcium handling	Arrythmias	Contraction	Sarcomere organization	Morphology	Publications
Myocardial infarction (cardiac injury)	Cardioids and cMTs	Cryoinjury or hypoxia + noradrenaline	Reduced OCR	Yes	Reduced amplitude	Yes	Reduced force	N/A	Cell death	[15,30,49]
Hypertrophic cardiomyopathy										
*MYBPC3*	EHTs	Patient cell line or introduced mutation with unrelated or isogenic control	Oxidative stress	No	Reduced/increased amplitude, prolonged transient, EAD-like events	No	Reduce/increase in force, duration, relaxation	Reduced organisation	Hypertrophy	[53,56,59,60]
*MYH7*	EHTs	Patient cell line or introduced mutation with unrelated or isogenic control	Oxidative stress	No	N/A	No	Increased force	Reduced organisation	Hypertrophy	[54,56]
*BRAF*	EHTs	Patient cell line with unrelated control	N/A	No	N/A	Yes	Increased force and relaxation, reduced duration	N/A	Hypertrophy	[109]
*PRKAG2*	EHTs	Patient cell line with isogenic control	Glycogen accumulation and oxidative metabolism	No	N/A	N/A	Increased force	N/A	Hypertrophy	[110]
*TNNT2*	EHTs	Introduced mutation	N/A	N/A	Increased amplitude	N/A	Increased force	Increased z-disk length	Hypertrophy	[111]
Arrhythmogenic cardiomyopathy										
*PKP2*	cMTs	Patient cell line and unrelated control	N/A	N/A	N/A	Yes	N/A	No	N/A	[33]
*DSP*	EHTs	Patient cell line and unrelated control	N/A	N/A	N/A	Yes	Increased diastolic length, diastolic and twitch stress and contractile shortening	N/A	Reduced number of desmosomes	[55]
Dilated cardiomyopathy										
*Titin*	EHTs	Patient cell line and unrelated control	N/A	N/A	N/A	N/A	Reduced force	Reduced organisation	N/A	[112]
*TNNT2*	EHTs	Introduced mutation	N/A	N/A	Reduced amplitude	N/A	Reduced force	Reduced z-disk length	Reduced cell size	[111]
Duchenne muscular dystrophy	Cardioids	Patient cell line and isogenic control	Increased adipogenesis	Yes	N/A	N/A	Reduced beat rate over time	N/A	Size differences	[22]
Arrhythmias (genetic variants)										
*KCNQ1* (LQTS1)	cMTs	Patient cell line unrelated control	N/A	N/A	N/A	Prolonged FPD	N/A	N/A	N/A	[31]
*KCHN2* (LQTS2)	EHTs	Patient cell line unrelated control	N/A	N/A	N/A	Prolonged APD	N/A	N/A	N/A	[42]
*CASQ2* (CPVT2)	EHTs	Patient cell line unrelated control	N/A	N/A	Arrythmias (double peaks)	Yes	N/A	N/A	N/A	[42]
Pregestational diabetes	Cardioids	Glucose and insulin	N/A	N/A	N/A	N/A	N/A	N/A	Size differences	[65]
Ebstein's anomaly	Cardioids	Introduced mutation or knock-out	N/A	N/A	Reduced transient duration	N/A	N/A	Reduced length	N/A	[15,66]
Other congenital diseases										
*Loss of HAND1*	Cardioids	Knock-out	N/A	N/A	N/A	N/A	N/A	N/A	Deficient cavity formation	[15]
*Loss of ISL1*	Cardioids	Knock-out	N/A	N/A	N/A	N/A	Delayed onset of contraction	N/A	Atrial and outflow tract malformations	[21]
*Loss of TBX5*	Cardioids	Knock-out	N/A	N/A	N/A	N/A	Lack of spontaneous beating	N/A	Impaired CM differentiation and all heart compartments are affected	[21]
*Loss of FOXF1*	Cardioids	Knock-out	N/A	N/A	N/A	N/A	Reduced contraction	N/A	Abnormal cavity formation, differentiation impairments (atrial & atrioventricu-lar canal)	[21]

EAD, early after depolarizations; FPD, field potential duration; APD, action potential duration; N/A, not applicable.

Besides ischemic cardiac injury, features of genetic cardiac diseases like cardiomyopathies also have been replicated in 3D cardiac models (Table 1). These myopathies, including arrhythmogenic, dilated and hypertrophic variants, arise from genetic mutations in key cardiac genes such as the sarcomeric genes *MYBPC3* and *MYH7*, and desmosomal genes *PKP2* and *DES*. While abnormal CM function and morphology are common features, these diseases exhibit variable penetrance and severity even within families with the same mutation. This means that patient management is challenging and sometimes empirical, with generic drug treatments being administered and leading to some patients not responding optimally to these drugs or even at all [50–52]. hPSC-derived 3D cardiac models provide an excellent opportunity to unravel disease mechanisms that may not be evident in (immature) 2D cultures, possibly because causative genes are not expressed. Paracrine signaling and spatial orientation of the different cardiac cell types in the 3D models can also influence manifestation of the disease phenotype. For example, the importance of stromal cells was highlighted in a study by Giacomelli et al. [33] in which cMTs consisting of wildtype CMs and diseased cFBs carrying a mutation in plakophilin-2 showed features of arrhythmogenic cardiomyopathy even though the CMs were normal. Moreover, it demonstrates a unique advantage of 3D cardiac models generated from pre-differentiated cells as these enable the combination of wildtype and diseased cell types, shedding light on the specific culprits in cardiac disease.

To investigate genetic cardiac diseases, researchers typically employ patient-derived hiPSC lines with genetically-repaired hiPSCs serving as isogenic controls, or introduce the mutation of interest into control hPSCs [22,53–56] (Table 1). These isogenic lines help ensure that observed phenotypes are mutation-specific and not simply due to genetic background differences [57]. EHTs are particularly useful for modelling cardiomyopathies, as they allow direct measurement of contractile forces [34,54,55,58]. For instance, EHT models of hypertrophic cardiomyopathy caused by mutations in *MYBPC3*, demonstrate both hypo- and hyper-contractile responses depending on the mutation, alongside other features like calcium handling abnormalities, hypertrophy and myofibrillar disarray [53,56,59,60] (Table 1). For other 3D cardiac cellular models in which contractile force cannot be directly measured, open-source or specialized software can be used to analyze pixel density or optical flow to obtain other contraction parameter readouts [22,61–64].

In addition to cardiomyopathies, genetic cardiac arrhythmias like long QT syndrome types 1 and 2 (LQT1 and LQT2), and catecholaminergic polymorphic ventricular tachycardia type 2 (CPVT2), have been modelled in both EHTs and cMTs (Table 1) [31,42]. For example, cMTs generated from hiPSCs with a *KCNQ1* mutation (LQT1) exhibited a prolonged field potential duration when compared with healthy controls, mimicking the expected patient electrophysiological abnormalities [31]. Similarly in EHT models of CPVT2, treatment with isoproterenol resulted in arrhythmias, mimicking the situation observed in patients in conditions of stress or exercise [42].

Cardiac organoids, or cardioids, are proving valuable for studying CHD where onset of these defects occurs during heart development. A recent publication by Lewis-Israeli et al. [65] used cardiac organoids to model pregestational diabetes-induced CHD, with the treated organoids showing morphological, structural and metabolic defects akin to pathological CHD phenotypes [65] (Table 1). Additionally, both cMTs and cardiac organoids have been shown to develop representative areas of the various compartments of the heart, such as the left and right ventricle, atria, outflow tract and atrioventricular canal [15,21,37,66–68] (Figure 1). Remarkably, cardioids can also specifically form cavities that are not simply a result of a hypoxic-induced necrotic core [15]. Formation of the different compartments of the heart can provide insights into specification of different cell types, for example by lineage tracing, as well as aiding in understanding region-specific disease effects. Indeed, Hofbauer et al. [15] showed in cardioids that knocking out *HAND1,* a gene linked to CHD, led to cavity formation deficiencies (Table 1). The fusion of organoids representing different heart compartments into structures called ‘assembloids’ further develops these models to better represent the architecture of the heart [21]. Mutations in genes such as *ISL1*, *TBX5* and *FOXF1* can cause cardiac compartment-specific defects [69]. When these genes were inactivated in organoids corresponding to specific heart regions, the effects mirrored the compartment-specific abnormalities observed in knock-out mouse embryos [21] (Table 1).

In summary, EHTs, cMTs and cardiac organoids/cardioids each possess unique features relevant to studying specific diseases, with no single model capturing all disease-specific phenotypes. The choice of model depends on the specific research question, with EHTs being optimal for force measurements and cardiac organoids/cardioids and cMTs offering high-throughput options. The EHT and cMT models also offer the flexibility to mix wildtype and diseased cell types. In sum, each model has its own pros- and cons and it is essential to choose the model best tailored to answer the biological question to hand.

## Pre-clinical testing of compounds

Despite significant strides in compound design and target discovery, drug development for cardiac diseases lags behind fields like oncology [70]. Moreover, cardiotoxic side effects of drugs are still sometimes missed until after a drug is released on the market. These issues are partly attributable to the high costs of screening for novel cardiac therapeutics and the limitations of established testing models. Therefore, despite considerable advances, there remains a clear need for new, physiologically-relevant, and low-cost high-throughput platforms to improve cardiac disease drug discovery and safety pharmacology [1]. 3D cardiac models that can be produced in large quantities and are more cost-effective compared with animal models, represent a promising solution.

Many studies developing 3D models for drug screening have primarily focused on investigating a limited amount of compounds in a proof-of-principle context. For example, the assessment of the contractile properties of cardiac tissues when exposed to E-4031 and isoproterenol [71], or analyzing the effects of the endoplasmic reticulum Ca^2+^ ATPase, thapsigargin, on biowire tissues [72]. Further efforts to evaluate larger libraries of new or existing compounds have mainly employed EHTs, typically through image-based analysis of contraction, action potentials or calcium transients [34,73,74]. An innovative approach, the Heart-Dyno method developed by Mills et al. [74], utilizes 96-well plates containing EHTs. The force can then be measured from contraction videos captured by high-content imaging systems. After initially screening 5000 compounds to identify those that could enhance 2D hiPSC-CM proliferation, 105 candidates were selected for further testing on EHTs. The proliferative effect of the compounds was determined by Ki-67 expression, while effects on contraction were via image-based contraction analysis. Using the Heart-Dyno platform the authors identified two small-molecules acting on the mevalonate pathway that increased proliferation without evident functional side effects [74]. Interestingly, the study also highlighted that compounds effective at inducing proliferation in 2D cultures failed to produce similar results in 3D models, underscoring the potential value of using more complex models for predicting drug efficacy and/or toxicity. It is important to note though that starting with 2D monocultures before moving to EHTs proved to be a cost-effective strategy to narrow down potential candidates. Additional efforts to utilize 3D cardiac models for drug discovery include a study by Mills et al. [32], which identified a BET inhibitor as a promising candidate for preventing SARS-CoV-2-induced cardiac dysfunction using EHTs.

Aside from the challenges in developing drugs to treat cardiac disease, the heart is also highly susceptible to the toxic side effects of medications. Patients can develop fatal arrhythmias due to cardiotoxicity and is the primary cause of attrition during drug development [75]. Accurate early prediction of cardiotoxic risks could notably reduce development costs and patient risks. Kofron et al. [76] developed a risk assessment platform using a voltage-sensitive dye in a high-content imaging system to measure action potentials and assess the cardiotoxic risk of pharmaceutical and environmental compounds. Validated with known high- and low-risk compounds, the platform shows promise, though it is important to note that organic dyes used can themselves be cytotoxic [77,78]. The use of less toxic alternatives, such as genetically encoded voltage and calcium indicators, have been employed in 2D hPSC-CMs [79–81], though current transfection methods are not very effective in 3D cardiac models. Given the advantages of these models, alternative transfection methods, like the use of lipid nanoparticles [82,83], may be required.

**Figure 2.**
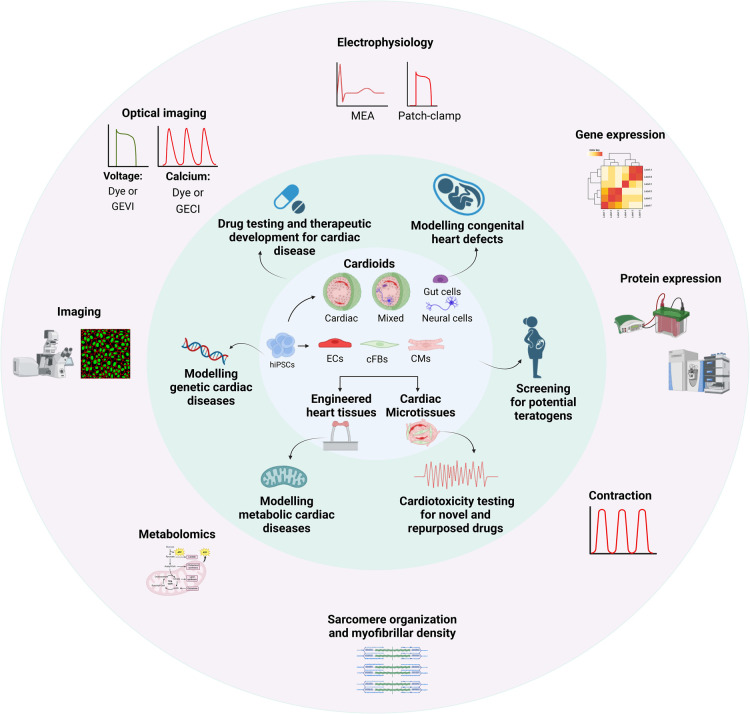
Overview of 3D cardiac cellular models and their applications. 3D cardiac cellular models derived from hPSCs are depicted, showcasing diverse approaches for generating these structures. Each model possesses distinct characteristics that may be beneficial for studying specific types of cardiac diseases (including genetic, congenital and metabolic disorders). Furthermore, these models can be in applied in pre-clinical testing to assess the safety and efficacy of treatments for cardiac disease and to evaluate the potential cardiotoxic risks of novel treatments. Functional assays that can be performed to study contraction, calcium handling and electrophysiology include optical imaging of voltage or calcium changes, MEA, patch-clamp electrophysiology, and analysis of brightfield contraction videos. Beyond physiological characterization, sarcomere organization and density, protein and gene expression profiles, and metabolic states can be assessed.

Overall, 3D cardiac models have broadened opportunities for pre-clinical drug testing and cardiotoxicity assessment (Figure 2). However, challenges related to standardization, qualification and validation persist, and are outlined in the following section.

## Addressing challenges in 3D cardiac models

### Variability, complexity and maturation

Technical and biological variability presents a significant challenge to the widespread implementation of 3D cardiac models, as their complexity can sometimes compromise consistency. For example, a study on kidney organoids showed high transcriptional correlation within batches but not between them, primarily due to differences in temporal maturation [84]. This underscores the need to identify whether these sources of variability arise from laboratory practices or are biological differences before they can be addressed. This is where automating the generation of the 3D cell models might help mitigate some of these issues, while careful experimental design, including the use of isogenic pairs, proper controls, and replicates, is essential. Cardiac organoids, with their less controlled cell type composition compared with EHTs and cMTs, might particularly benefit from automated differentiation techniques, such as the use of automated liquid handling systems which could reduce overall variability. Such benefits were observed when automation was applied to the generation, maintenance and analysis of midbrain organoids, with more uniform morphology and reduced intra- and inter-batch variability in gene expression and cell composition [85].

The relative immaturity of CMs in some cardiac organoid models also still presents obstacles for studying adult diseases. However, several methods have been proposed to address this issue [86–88], with simple strategies like using a maturation media being reported [37,74,89]. Volmert et al. [37], for example, experimented with mimicking metabolic and hormonal developmental changes by supplementing the culture medium with fatty acids, L-carnitine, T3 hormone, ascorbic acid and IGF-1. Nanoengineering approaches, involving the integration of nanoparticles or nanowires to improve contractility and engraftment potential, are also being explored [89,90]. Thus, research efforts focused on strategies to reduce variability and enhance the reproducibility of these models to ensure their robustness is ongoing.

### Incorporating immune cells

Inflammation is an underlying trigger for many diseases, including those of the heart. Immune cells, such as macrophages, play important and dual roles in MI. Initially, they are responsible for removal of dead cell debris and tissue repair, while in later stages they are involved in fibroblast activation, resulting in myofibroblast conversion and fibrosis development [91]. Immune cells are also a vital part of tissue homeostasis. In the heart, macrophages contribute to energy homeostasis, electrical conduction and the establishment and modulation of the lymphatic system [91–94]. Incorporating immune cells into these 3D cell models could thus facilitate the modelling of additional disease aspects. Similar strategies have been employed in other organoids, such as brain and gut, where either microglia or cytotoxic T lymphocytes were introduced to study infection in these tissues [95,96]. Typically, these immune cells are blood-derived and are subsequently mixed with the organoid [97]. However, differentiating the immune cells from hiPSCs and integrating them into the 3D model would offer greater control over their numbers and types, and potentially even allow them to be isogenic [98,99]. Another approach could involve co-differentiating immune cells during cardioid differentiation, as was recently demonstrated with intestinal organoids [100], although challenges will remain in controlling the type and proportion of immune cells per cardioid. Nevertheless, incorporating immune cells or introducing inflammatory triggers in the 3D cardiac models could provide new insight into the role of immune cells in disease, homeostasis and development.

### Electrophysiology

Electrophysiological measurements in 3D cardiac models presents certain challenges. Techniques like microelectrode arrays (MEA) and patch-clamp electrophysiology, originally designed for 2D monolayer cultures, have been adapted for use with 3D cultures [21]. For patch-clamp analysis, sharp electrodes offer a way to probe the electrical properties of the cells within for example cMTs, although interpreting the data is more complex due to the influence of the surrounding 3D matrix and cell-to-cell interactions. Alternatively, the 3D tissues can be dissociated and the cells plated in 2D for measuring action potentials and ion channel currents on isolated CMs [21,31]. Innovative methods, like the use of ‘shell’ MEAs that fold around brain organoids, could be applicable to cardiac organoids or cMTs [101]. Other bioengineering approaches have incorporated electrodes within the organoids for both pacing and measurement purposes [102]. Photoelectrochemical imaging with light-addressable potentiometric sensors has also been used to achieve high sensitivity action potential measurements [103].

### High-throughput readouts

While the developments described above would allow electrophysiological measurements of the 3D models, these methods are slow and likely to remain low-throughput, hindering the transition of some models to pre-clinical testing. For high-throughput phenotyping and drug screening, cardioids and cMTs are suitable but require scaled-up production and standardized, automated readouts. Functional assessments, such as calcium transients and action potentials, can be visualized using organic dyes as mentioned earlier, with data automatically collected in high-content imaging systems, although the potential influence of these dyes on the observed effect needs to be considered [12]. Contraction analysis, too, can be automated using brightfield contraction videos [34,64].

There are also challenges regarding imaging 3D structures for determining marker expression, cellular composition and internal features. While new clearing techniques enable imaging of entire 3D structures, it remains low-throughput and requires data pipelines capable of handling large files [104,105]. A novel high-throughput imaging method using microfabricated chips, termed ‘JewWells’, has shown promise, allowing the imaging of up to 300 organoids per hour and using deep learning tools to analyze organization within the organoids [106]. Another method to study structure is optical coherence tomography, which can be employed to study cavity formation and beating patterns over time [107].

Single cell dissociation of 3D cardiac models for analysis can also be problematic, with certain cell populations, like ECs, being hard to recover and being underrepresented in downstream assays such as flow cytometry and single-cell RNA-sequencing [16]. Developing improved dissociation methods is essential, particularly for disease models where ECs may play a crucial role. Additionally, the development of faster methods to extract information about cell states within the 3D structure could benefit pre-clinical testing. For example, cell-free DNA released by cardiac organoids in the culture medium was shown to reflect specific stages of differentiation and disease markers were also increased after treatment with cardiotoxic drugs [108]. This suggests that detecting cell-free DNA could enhance throughput in screenings and in real time monitoring. In summary, while many new techniques are being developed to improve standardization and high-throughput data collection from 3D cardiac models for pre-clinical testing, challenges in various aspects of their use and analysis remain an active area of research.

## Perspectives

- Significant challenges remain to be addressed to fully leverage the potential of 3D cardiac models. These include high variability, developmental immaturity, absence of immune cells and the fact that large scale methods for assessing functional characteristics are still in their early stages. Despite these obstacles, 3D cardiac models offer promising low-cost, high-throughput platforms for pre-clinical testing. Given the high costs and sometimes limited relevance of animal models to human cardiac diseases, these cellular models offer a more human-centric approach. They have the potential to not only supplement but also partially replace animal models, especially in evaluating the efficacy of treatments for human heart conditions.
- With ongoing technical and bioengineering advancements, human 3D cardiac models are becoming increasingly capable of replicating a wide range of disease phenotypes *in vitro*. Further improvements in standardization are expected to facilitate the broader adoption and acceptance of these models, also by regulatory agencies.
- In the near future, the use of human 3D cardiac models in pre-clinical testing is poised to become more sophisticated and efficient. The integration of robotics for organoid generation, and automated systems for their characterization and functional analysis will streamline the testing process. This advancement will not only enhance the precision and reliability of pre-clinical testing, but also significantly expedite drug development and safety assessment.

## Abbreviations

3D: 3-dimensional
2D: 2-dimensional
cFB: cardiac fibroblast
CHD: congenital heart defects
cMT: cardiac microtissue
CPVT2: catecholaminergic polymorphic ventricular tachycardia type 2
ECM: extracellular matrix
EC: endothelial cell
EHT: engineered heart tissue
hPSC: human pluripotent stem cell
hESC: human embryonic stem cell
hiPSC: human induced pluripotent stem cell
hPSC-CM: hPSC-cardiomyocyte
LQT1/LQT2: long QT syndrome 1 and 2
MEA: microelectrode arrays
MI: myocardial infarction
GEVI: genetically encoded voltage indicator
GECI: genetically encoded calcium indicator
